# Comprehensive analysis of RNA-seq data reveals the complexity of the transcriptome in *Brassica rapa*

**DOI:** 10.1186/1471-2164-14-689

**Published:** 2013-10-07

**Authors:** Chaobo Tong, Xiaowu Wang, Jingyin Yu, Jian Wu, Wanshun Li, Junyan Huang, Caihua Dong, Wei Hua, Shengyi Liu

**Affiliations:** 1 Key Laboratory of Biology and Genetic Improvement of Oil Crops, Ministry of Agriculture, P.R. China, Oil Crops Research Institute of the Chinese Academy of Agricultural Sciences, Wuhan 430062, China; 2 Key Laboratory of Biology and Genetic Improvement of Horticultural Crops, Ministry of Agriculture, P.R. China, Institute of Vegetables and Flowers, Chinese Academy of Agricultural Sciences, Beijing 10081, China; 3 Beijing Genome Institute-Shenzhen, Shenzhen 518083, China

**Keywords:** *Brassica rapa*, RNA-seq, Alternative splicing, Transcriptome

## Abstract

**Background:**

The species *Brassica rapa* (2n=20, AA) is an important vegetable and oilseed crop, and serves as an excellent model for genomic and evolutionary research in *Brassica* species. With the availability of whole genome sequence of *B. rapa*, it is essential to further determine the activity of all functional elements of the *B. rapa* genome and explore the transcriptome on a genome-wide scale. Here, RNA-seq data was employed to provide a genome-wide transcriptional landscape and characterization of the annotated and novel transcripts and alternative splicing events across tissues.

**Results:**

RNA-seq reads were generated using the Illumina platform from six different tissues (root, stem, leaf, flower, silique and callus) of the *B. rapa* accession Chiifu-401-42, the same line used for whole genome sequencing. First, these data detected the widespread transcription of the *B. rapa* genome, leading to the identification of numerous novel transcripts and definition of 5'/3' UTRs of known genes. Second, 78.8% of the total annotated genes were detected as expressed and 45.8% were constitutively expressed across all tissues. We further defined several groups of genes: housekeeping genes, tissue-specific expressed genes and co-expressed genes across tissues, which will serve as a valuable repository for future crop functional genomics research. Third, alternative splicing (AS) is estimated to occur in more than 29.4% of intron-containing *B. rapa* genes, and 65% of them were commonly detected in more than two tissues. Interestingly, genes with high rate of AS were over-represented in GO categories relating to transcriptional regulation and signal transduction, suggesting potential importance of AS for playing regulatory role in these genes. Further, we observed that intron retention (IR) is predominant in the AS events and seems to preferentially occurred in genes with short introns.

**Conclusions:**

The high-resolution RNA-seq analysis provides a global transcriptional landscape as a complement to the *B. rapa* genome sequence, which will advance our understanding of the dynamics and complexity of the *B. rapa* transcriptome. The atlas of gene expression in different tissues will be useful for accelerating research on functional genomics and genome evolution in *Brassica* species.

## Background

The species *Brassica rapa* (2n=20, AA) includes several subspecies providing human nutrition in the form of leafy, root and stem vegetables and edible oil. It also represents the origin of the *Brassica* 'A’ genome and contributes to other cultivated oilseed crops of *Brassica* allopolyploids: *B. napus* (AACC) and *B. juncea* (AABB) [1]. Therefore, *B. rapa* has great potential as a model for genomic and evolutionary research in *Brassica* species. Over the past decade, a growing number of genomic resources for *B. rapa* have become available [2-5], in particular the whole genome sequence of *B. rapa* accession Chiifu-401-42, an inbred Chinese cabbage line, which was published in late 2011 [6].

With the availability of the *B. rapa* genome sequence [6], it is essential to further identify and determine the activity of all functional elements on a genome-wide scale. For this purpose, a comprehensive analysis of the transcriptome is required to reveal potentially transcribed regions and understand expression patterns of the entire *B. rapa* gene model sets across tissues. High-throughput RNA-seq technology represents a powerful and cost-efficient tool for transcription profiling [7]. First, it can detect and quantify gene expression with digital measurements, and is especially sensitive for low-expressed genes [8]. Second, it allows researchers to improve gene annotation by extending transcriptional boundaries of genes and permits the discovery of novel genes or transcripts [9]. Third, the most attractive advantage is that RNA-seq can survey alternative splicing (AS) events at single nucleotide resolution [10-13]. In addition, RNA-seq data show a high level of reproducibility in both technical and biological replicates [14].

In this study, we generated RNA-seq data from six different tissues (root, stem, leaf, flower, silique and callus) of the *B. rapa* accession Chiifu-401-42, which was the reference line used for whole genome sequencing [6]. By performing a comprehensive analysis of these RNA-seq data from various tissues, our goals were: (i) to identify actively transcribed regions in the *B. rapa* genome, including annotated coding sequences and novel transcribed regions (NTRs); (ii) to unveil the transcriptome dynamics of *B. rapa* gene models, and identify candidate genes that are conserved, specific, or show correlated expression across tissues; and (iii) study the AS of *B. rapa* genes and investigate whether 'AS-preferred’ genes are enriched in certain functional categories or are associated with particular gene structure, such as short introns.

## Results and Discussion

### Generation, mapping, and assessment of RNA-seq reads

Paired-end RNA-seq reads of 90 bp in length were generated from six major organs or tissues of *B. rapa* including root (two samples), stem, leaf (two samples), flower, silique and callus. The base quality of RNA-seq reads was checked and analyzed using FastQC (http://www.bioinformatics.babraham.ac.uk/projects/fastqc/) (in Additional file 1: Figure S1 and Figure S2). We used relatively stringent criteria for quality control by removing the pair-end reads containing Ns or those where the number of bases whose PHRED-like score was less than 20 exceeded 10% (see *Methods*, in Additional file 1: Figure S3, in Additional file 2: Table S1). For eliminating the errors from the mis-priming of primers in RNA-seq experiments, the first nine base pairs of reads were trimmed before read alignment. Using the software TopHat2 [15] and allowing an at most 2-bp mismatch, we obtained over 219 million uniquely mapped reads for subsequent analyses (in Additional file 2: Table S2). Seventy-four percent of uniquely mapped reads were contiguously mapped back to the *B. rapa* v1.5 genome sequences, and the others were splice-mapped to, and spanned potential splice junctions of the genome (in Additional file 1: Figure S4, in Additional file 2: Table S2). The uniquely mapped reads covered over 80 million bases of the total genomic bases, with an average read-depth of 26–75 per base across different samples (in Additional file 2: Table S2). Around 81% of uniquely mapped reads confirmed the expression of ~40 Mb out of a total of 50 Mb of annotated *B. rapa* coding sequence (CDS). The remaining ~16% and ~3% of uniquely mapped reads were aligned to another ~40 million genomic bases in intergenic and intronic regions, respectively (in Additional file 2: Table S2), implying that large amounts of actively transcribed regions remain unidentified in the *B. rapa* genome. The average read depth of coverage in CDS, introns and intergenic regions are shown in Additional file 1: Figure S4. As expected, the read depth in intergenic regions and introns was lower than that in exons, which correlated with the subsequent observation in this study that novel transcripts discovered from these regions are commonly expressed at low levels. The read depth seemed to be distributed relatively evenly along the whole body of genes (in Additional file 1: Figure S5 and Figure S6), reflecting no introduction of obvious bias during randomly primed reverse transcription and subsequent RNA sequencing. The two samples of root and leaf tissues from different individual plants both showed a high degree of concordance in gene expression of log2-transformed fragments per kilobase of transcript per million fragments mapped (FPKM) values (R-squared of 0.94 for both comparisons, in Additional file 1: Figure S7).

### RNA-seq data improved the annotation of the *B. rapa* genome

The transcribed regions/units were constructed independently in individual tissues and then merged into a final dataset containing 37,002 transcripts (in Additional file 2: Table S3). 33,598 transcripts from this dataset completely (43%) or partially (57%) overlapped with 30,322 annotated gene models in *B. rapa*, and the remaining transcripts results in the identification of 3,450 novel transcribed regions (NTRs) in the *B. rapa* genome (in Additional file 2: Table S4).

**Figure 1:**
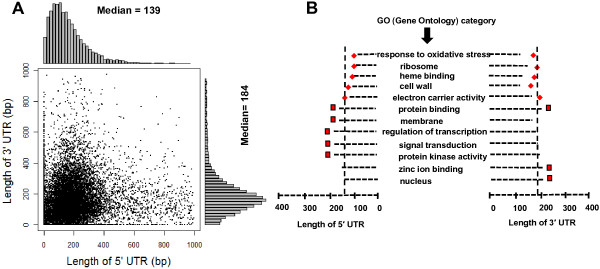
**UTRs analysis of *B. rapa *genes. (A)** Scatter-plot and histogram showing the length distributions of identified 3*'* and 5*'* UTRs based on RNA-seq data. **(B)** Transcripts with significantly larger (diamonds) or smaller (square) UTRs for selected GO categories. Vertical dashed lines represent median UTRs length.

The *B. rapa* gene models (v1.5) were annotated only using open reading frames (ORFs), and thus the 5′ and 3′ boundaries of untranslated regions (UTRs) have not been defined. Here, through globally comparing length-complete transcripts with corresponding *B. rapa* gene models, we defined the 5′-boundary regions of 12,879 transcribed genes and the 3′-boundary regions of 12,485 transcribed genes (in Additional file 2: Table S5)*.* The median 5′- and 3′-UTR lengths determined by the RNA-seq data were 139 and 184 nucleotides, respectively (Figure 1A). The estimated length of 5′-UTRs in *B. rapa* is longer than that in the closely related model species, *Arabidopsis thaliana* (median = 88); however, their 3′-UTRs are similar (median = 184) [16].

Gene ontology (GO) categories with significantly longer or shorter UTRs were obtained by comparing the lengths of all UTRs of each GO category with total UTR length (Wilcoxon Rank Sum test, P < 0.05). The transcripts/genes related to regulatory signals and binding domains seem to have long 5′ and 3′-UTRs, as exemplified in the transcripts/genes encoding protein kinases, membrane proteins, and signal transducers (Figure 1B). By contrast, ribosomal genes seem to have short 5′-UTRs. This observation is consistent with previous reports in other organisms [11,17]. In addition, we scanned a small upstream ORF (uORF) of start codons, and found that 4,147 (~29% of the total) genes contain a uORF (10–50 nt) in the 5′-UTR region (in Additional file 2: Table S6). The importance and mechanism of uORFs as regulatory factors for gene expression have been revealed clearly in recent years [18,19]; therefore, further analyses of these novel UTRs should greatly aid research towards identifying all known regulatory elements in the *B. rapa* genome.

### Discovery of novel transcripts

We identified 3,450 NTRs that are not linked to any annotated gene models in *B. rapa* (in Additional file 2: Table S4). Over 41.2% (1,422) of the NTRs have an ORF greater than 100 amino acids as predicted by Augustus [20], and 802 of them contain pfam protein domains [21]. Additionally, 166 NTRs have sequence similarity (greater than 100-nucleotide match and 60% identity) to transposable elements (TEs) in Repbase (v16.10), reflecting substantial TE activity in the *B. rapa* genome. Only ~20% of them are supported by expressed sequence tags (ESTs) (using blat, more than 95% match in identity and coverage), despite using 214,106 ESTs from the National Center for Biotechnology Information (NCBI) database. To validate the predicted novel transcripts, we randomly selected six of them for reverse transcription polymerase chain reaction (RT-PCR) analysis. All six were confirmed to have transcriptional activity in at least one tissue (in Additional file 1: Figure S8).

**Figure 2:**
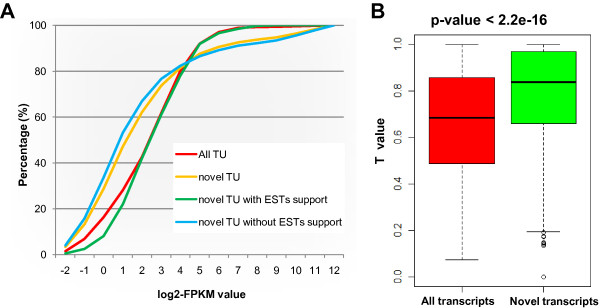
**Expression pattern of novel transcripts. (A)** A comparison of expression levels between novel and general transcripts, as well as between novel transcripts with EST support and those without EST support. **(B)** Tissue-specific expression profiles of novel transcripts.

We quantified the expression of the constructed transcripts using FPKM values [22], and then compared the novel transcripts with annotated ones in terms of expression level and tissue breadth of expression. First, the FPKM cumulative distribution revealed that these novel NTRs had much lower transcription levels than known transcripts (genes) (Figure 2A). This agreed with another comparison that showed that novel NTRs without EST support were expressed at a significantly lower level than those with EST support (Figure 2A). This feature of low expression level may explain why these novel NTRs have not been annotated before, but were detected effectively by RNA-seq technology. Second, we found that novel NTRs showed narrow tissue breadth compared with annotated genes, as shown by the T value which scores a value between 0 for housekeeping genes and 1 for tissue-specific genes [23] (Figure 2B, Wilcoxon Rank Sum test, P < 2.2e-16). The feature of preferential organ-specific expression may be another reason for these genes not being annotated in the *B. rapa* genome [24]. It should be noted that some tissue-specific NTR may not be real, because we could not collect all diverse tissues or sufficient replicates; however the conclusions were based on the comparison of tissue breadth between NTRs and annotated genes in the same background.

These NTRs were difficult to discover previously because of their low-level and organ-specific expression; however, high-throughput sequencing technology and individual organ libraries permitted their discovery. Further analysis showed that highly expressed and poorly expressed novel transcripts were relatively equally detected in silique and callus tissues, unlike in other tissues, where poorly expressed novel transcripts were preferentially discovered (in Additional file 1: Figure S9). We speculated that because the current *B. rapa* genomic annotation is mainly based on ESTs collected from normal tissues, transcripts preferentially expressed in these two relatively specialized tissues could have remained unannotated. Thus, generating RNA-seq data from more diverse tissues from different developmental stages or environmental conditions will be helpful for improving the annotation of the *B. rapa* genomic sequence.

### Tissue dynamics of *B. rapa* transcriptomes

**Figure 3:**
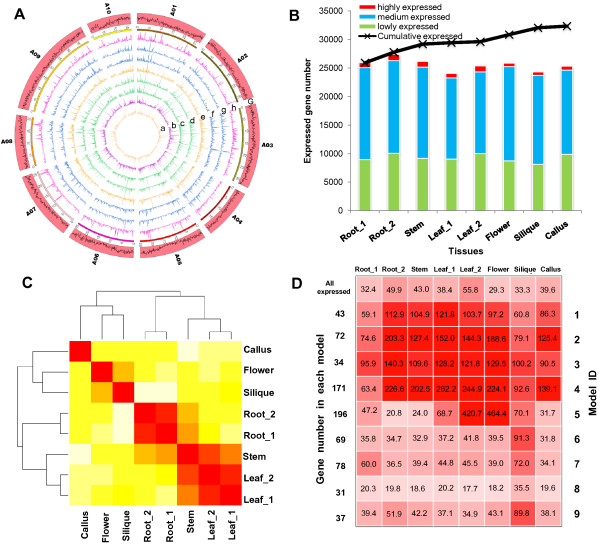
**Dynamics of *B. rapa *gene expression in tissues. (A)** The expression levels, defined as numbers of reads per kilobase per million mapped reads within each 100 Kb window, are shown, along *B. rapa* ten chromosomes (1–10) in each tissue. a: Callus, b: Root_1, c: Root_2, d: Stem, e: Leaf_1, f: Leaf_2, g: Flower, h: Silique; G: Gene density within each 100 Kb non-overlapping window represented as log2-tranformed total length of genes. **(B)** The number of highly (FPKM > 50), medium (5 < FPKM ≤50), and lowly (FPKM ≤ 5) expressed genes in each tissue. The black line shows the cumulative expressed gene number as the tissue number increased. **(C)** The dendrogram of tissue transcriptomes based on clustering of log2-transformed FPKM values of constitutively expressed genes. **(D)** Nine modules (ID: 1–9) from WGCNA analysis. The mean gene FPKM value within each module is marked by figures in blocks, and also represented as the color (red) depth of the block. The mean FPKM values of all expressed genes in each tissue are shown in the first row of blocks for comparison. The detailed information of genes in each module is provided in Additional file 2: Table S10.

The read depth of coverage for each *B. rapa* genomic location was calculated across all tissues. This information is presented in .wig files, which, together with raw RNA-seq data have been submitted to the GEO database (accession number GSE43245). Transcriptome characteristics including tissue specificity, and conserved or novel transcripts in the genome, can be viewed with implemented Integrative Genomics Viewer (IGV) or other visualization tools (Figure 3A, in Additional file 1: Figure S10).

For each gene model, it was considered expressed if lower boundary of the FPKM values for the 95% confidence interval of the abundance of gene was greater than zero (in Additional file 2: Table S7). 32,335 genes (78.8% of the total 41,020 annotated genes) were detected as expressed in at least one tissue, of which 18,876 genes were constitutively expressed in all tissues (Figure 3B). The clustering of log2-transformed FPKM values of constitutively expressed genes shows the transcriptomic relationship between tissues as a dendrogram: (((leaf, stem), root), (flower, silique)), callus) (Figure 3C).

We obtained a dataset containing 867 highly and stably expressed genes representing tissue-conserved expressed genes whose FPKM values are greater than 50 in every tissue (in Additional file 2: Table S8). This dataset contains potential useful housekeeping genes such as *glyceraldehyde-3-phosphate dehydrogenase*, *actin*, *ubiquitin*, *tubulin* and *elongation factor 1-a*, *ribosomal protein*, *calmodulin, glutathione peroxidase* and *G-box regulating factor.* For tissue-specific expressed genes, we observed some significantly meaningful gene groups in flower and silique tissues (in Additional file 2: Table S9). Many genes encoding lipid transfer proteins, oleosin, glycosyl hydrolase, lipase and transferases involved in protein amino acid glycosylation and phosphorylation, biosynthesis of flavonoids, biosynthesis of long chain fatty acids, and cellulose biosynthetic process were specifically expressed and functional in siliques. With respect to flower-specific gene expression, rapid alkalinization factor, pectate lyase, pollen Ole e 1 allergen and extensin family protein, pectinesterase family protein, arabinogalactan-protein involved pollen tube growth, invertase/pectin methylesterase inhibitor, pectate lyase, and S1 self-incompatibility protein-related were identified.

To identify highly correlated groups of genes (called 'modules’), we conducted weighted gene correlation network analysis using the R-package [25,26]. After inspection of functional annotation of genes (inferred from the most similar gene in *A. thaliana*) in each module, we identified some meaningful co-expressed genes (Figure 3D, in Additional file 2: Table S10). First, four modules (ID: 1–4) abundant in ribosomal genes (40s or 60s subunit) were highly expressed across all tissues. Second, one module (ID: 5, 196 genes) is nearly exclusively committed to photosynthesis, with predominant expression of genes in leaf tissue encoding photosystem I and II subunit proteins, chlorophyll I and II binding proteins, rubisco subunit and activase, and other genes involved in chlorophyll biosynthesis, the photosynthetic electron transport chain, carbon utilization by fixation of carbon dioxide and light harvesting*.* We noticed that a similar group of co-expressed genes was reported in the maize leaf developmental transcriptome [27], implying that indispensable co-expression of these genes is required for leaf photosynthesis. Third, three modules (ID: 7–9) were significantly enriched for protein kinases and transcription factors (TFs) (146 genes), which were highly expressed in flowers.

### Identification of splice junctions and alternative splicing (AS) events

A total of 156,516 unique splice junctions (read depth of each junction ≥ 3 in each sample) were detected using TopHat [15] (in Additional file 2: Table S11); 122,693 (78.4%) of them confirmed 74.1% of all known 165,564 exon-exon junctions in the *B. rapa* genome. Examination of the dinucleotides at the splicing borders showed that 98.4% of them had GT-AG splicing junctions (in Additional file 1: Figure S11).

These predicted junctions allowed us to further define four main types of AS events as intron retention (IR), exon skipping (ES), alternative 5′ splice site donor (A5SS), and alternative 3′ splice site acceptor (A3SS) in each tissue [24]. A total of 15,432 AS events in all tissues, representing the four known types of AS, were identified in 7,668 genes (Table 1, in Additional file 2: Table S12). Only 26,063 of the 32,335 expressed genes contained two or more exons; therefore, it was estimated that about 29.4% (7,668/26,063) of the intron-containing genes are alternatively spliced. We also found that about 42.6% of them underwent multiple AS events, producing a variety of transcripts from a single gene (in Additional file 1: Figure S12), illustrating the high complexity of the transcriptome.

**Table 1:** **Predicted alternative splicing (AS) events in *B. rapa***

**AS type**	**AS events**	**Genes with AS event**	**AS events shared by more than two tissues**
IR	10275	4648	3568
A3SS	2741	2389	1076
A5SS	1170	1076	383
ES	1246	1107	329
Total	15432	7668	5356

**Figure 4:**
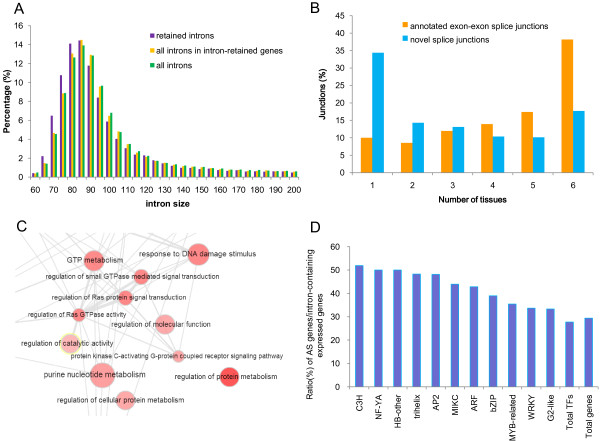
**Alternative splicing (AS) events in *B. rapa*. (A)** Size distribution of AS-retained introns (purple) compared with introns in IR genes (yellow) and introns in general (green). **(B)** The tissue breadth for novel and annotated exon-exon splice junctions. The percentage of splice junctions shared by different number of tissues was plotted. **(C)** Some AS-enriched GO categories associated with regulation and signal transduction. **(D)** TFs subfamilies with high AS frequency.

Similar proportions of AS genes have been observed in rice (33%) and maize (37%); however, the proportion detected in *B. rapa* was lower than that in *A. thaliana* (42% and 61%) [24,27-29]. This difference in the distribution of AS genes may be explained by higher numbers of EST/RNA-seq that are available for the model species *A. thaliana*. We believe that the figures presented here represent an underestimate and more AS events in *B. rapa* remain to be revealed, because AS is estimated to occur in 95% of intron-containing genes in humans [30,31]. In addition, we observed that IR is the most common event and the use of alternative 3′ splice sites is more than two-fold more frequent than the use of alternative 5′ splice sites. This pattern, particularly the abundance of IR, is conserved in diverse plants such as *Medicago truncatula*, *Populus trichocarpa*, *A. thaliana*, *Oryza sativa*, *Chlamydomonas reinhardtii*, and *Brachypodium distachyon*[32-35]. The prevalence of IR in *B. rapa* and other plants supports an intron-definition mechanism for pre-messenger RNA splicing [36]. We found that the average size of retained introns was significantly shorter than that of introns in general in *B. rapa* (mean 136 bp *vs*. 210 bp; Wilcoxon Rank Sum test: P < 2.2e-16; Figure 4A). This observation in *B. rapa* further supports the hypothesis that plants always have small introns compared with animals, which have large introns and therefore use an exon-definition mechanism, resulting in higher rates of ES [10,31,37].

We further investigated the tissue distribution of predicted splice junctions (Figure 4B). We divided all 165,564 read-supported splice junctions into two classes: the 122,693 known or annotated genomic exon-exon splice junctions and the remaining 33,823 novel splice junctions. It could be assumed that the former represent the junctions used for normal transcription, whereas the latter (novel splice junctions) always reflect the existence of AS and produce novel splice variants of transcripts [30,31]. We observed that each tissue dataset contributed between 50% and 78% of the read-supported known exon-exon splice junctions and the majority (90%) of them was detected to be shared by more than two tissues. This represents the normal and most frequent use of constitutive transcripts or isoforms in different tissues. For novel splice junctions, 65.6% of them were shared by more than two tissues and the remaining were detected in only one tissue representing tissue-specific or tissue-restricted splice variants (Figure 4B). It may be that AS events appear to be tissue-specific because they are rare, and more samples and replicates must been included to confirm the estimation for the tissue-specific splice junctions. However, a larger proportion of novel splice junctions are commonly detected in multiple tissues, indicating the prevalence of AS across tissues in the *B. rapa* genome.

### 'AS-preferred’ genes

Analysis of GO enrichment for AS genes, using all *B. rapa* genes as the background, revealed that GO categories enriched with AS genes were associated with signal transduction, regulation, response, binding and catalytic activity (Figure 4C, in Additional file 2: Table S13). It is worth noting that a GO category (GO: 0032318: regulation of Ras GTPase activity) and its paternal GO categories were significantly abundant in AS genes. Ras/small GTPases generally play a role in signal transduction as molecular switches for a variety of cellular signaling events [38], and AS may be essential for these genes to function in regulation and signal transduction.

Considering the possible AS effect on 'regulation’-related genes, we conducted a systematic survey of AS events that occurred in genes encoding TFs. First, based on annotated TF genes in *A. thaliana* (http://arabidopsis.med.ohio-state.edu/AtTFDB/), we identified 2,361 *B. rapa* genes and classified them into 57 different TF families through homology searching and domain identification (in Additional file 2: Table S14). Second, about 89% of TF genes showed expression in at least one tissue and 27.7% of those underwent AS. Subsequently, we examined the ratio of AS genes in each TF subfamily, and identified 11 TF subfamilies, such as ARF, AP2, MIKC, C3H and MYB-related, that have higher AS frequencies than others (Figure 4D). We found that previously reported AS events in TF genes, such as in CCA1/LHY-like MYB [39], R2R3-type MYB [40], MIKC-type MADS [41], bZIP [42], and NAC [43], almost all belong to these 'AS-preferred’ TF subfamilies. As suggested in metazoan [44], the above observation shows that alternative splicing may serve as an important and prevalent mechanism for these TF-encoding genes to form protein complexes that function in transcription in *B. rapa*.

Intriguingly, these 'AS-preferred’ genes were also those that were more likely to have been retained in mesopolyploid *B. rapa* during the diploidization process following a recent whole genome triplication that occurred in a *Brassica* ancestor ~13 million years ago [6]. In other angiosperms TFs and signal transducer genes are preferentially retained as duplicates after whole genome duplication (WGD) events [45-48]. This was popularly explained by the gene balance hypothesis [49], which suggests that genes participate in the formation of macromolecular complexes, or are involved in more interaction or regulation are dosage sensitive and are thus expected to be retained more often after WGD [50]. AS is able not only to remove functional domains to produce non-functional transcripts, thus regulating gene dosage [44,51], but also causes subfunctionalization between WGD-derived paralogous genes [52,53]. Thus, the observation that over-retained genes always have a high frequency of AS suggested that alternative splicing, as a fundamental aspect of the expression of many genes, may be a form of divergence of duplicated genes and thus contributed to their retention, although the possibility that AS and retention may are parallel and independent phenomena driven by a third selective factor, could not be excluded.

## Conclusions

In conclusion, the RNA-seq data revealed the extent of transcription of the *B. rapa* genome, and simultaneously improved the existing gene annotation. The identification of numerous novel NTRs, commonly poorly or tissue-specifically expressed, led to the proposal that further transcriptome data from high-throughput platform will unveil previously unidentified functional regions of the *B. rapa* genome. A complete picture of the transcriptome across the major organs of *B. rapa* was provided, including candidate tissue-specific, tissue-conserved, or tissue-correlated expressed genes. The comprehensive analysis of AS and splice junctions increased our understanding of the complexity of the *B. rapa* transcriptome.

## Methods

### Organ collection and RNA sequencing

Six tissues of *B. rapa* accession Chiifu-401-42, including callus, root, stem, leaf, flower, and silique, were prepared for mRNA extractions. Plants were grown under greenhouse conditions at 22°C. Callus tissue was obtained from tissue culture. Root, stem and leaf tissues were collected from seven-week old plants. Two samples of root and leaf tissues were generated from different batches of plants. Flower tissue was obtained from blooming plants on the same day without the floral shoot. Silique tissue was generated from 15-day plant after pollination. We constructed an individual cDNA library with insert sizes of 200 bp for each sample, and sequenced them on the Illumina Hiseq 2000 platform. The libraries were sequenced for paired-end reads of 90 bp. All RNA-seq raw data from this study have been submitted to the NCBI Gene Expression Omnibus (http://www.ncbi.nlm.nih.gov/geo/) under accession no. GSE43245.

### Mapping short reads and constructing transcripts

We used FastQC (http://www.bioinformatics.babraham.ac.uk/projects/fastqc/) to check and visualize the quality of the RNA-seq reads (in Additional file 1: Figure S1 and Figure S2). The average read quality was from 36 to 38 in different sample and the first nine base pairs of the reads, which showed unstable base composition given by the percentages of four different nucleotides (A, T, C, and G), were removed. We use the tools from the NGSQCToolkit (v2.3) [54] to remove the pair-end reads containing Ns or those where number of bases whose PHRED-like score was less than 20 exceeded 10% (in Additional file 2: Table S1). We calculated the sequence similarity of 10447 duplicated genes from the *B. rapa* triplicated genomes to evaluate the possible false reads for mapping (in Additional file 1: Figure S3). The *B. rapa* genome sequence (v1.5) was downloaded from the BRAD database (http://brassicadb.org/) for mapping and analysis We trimmed the first 9bp of filtered reads before read mapping using TopHat (v 2.0.9.) [15]. The uniquely mapped reads were used for subsequent analysis, including transcripts construction, quantification of gene and transcript expression, and identification of splice junction and AS events. The transcripts were constructed and quantified the expression FPKM values of transcripts in each sample by Cufflinks (v2.1.0) and merged into a final comprehensive set of transcripts using Cuffmerge.

### Tissue breadth of transcripts expression

The tissue specificity index T was calculated according to the formula developed by Yanai *et al.*[23].

$$\tau i=\frac{\sum_{j=1}^{n}(1-S\left(i,j\right)/S\left(i,\max\right))}{n-1}$$

In the formula, n is the number of surveyed *B. rapa* tissues. S (i, j) is the FPKM values of i gene in j tissue, and S (i, max) is the highest FPKM values of gene i in the n tissues.

### Weighted Correlation Network Analysis (WGCNA)

Gene expression FPKM values were log2 transformed before being processed through the WGCNA R package [26]. Co-expression analysis was performed to identify modules of highly correlated genes by the suggested protocol [25]. We used the test to select the best parameters (softpower = 26). All other parameters were used with the default values.

### Alternative splicing prediction

We used TopHat software [15] to identify splice junctions, then filtered junctions with read support of less than three. Each junction was searched for putative donor sites and acceptor sites inside intron regions. We predicted four types of AS events including exon skipping (ES), intron retention (IR), alternative 5′ splice site (A5SS) and alternative 3′ splice site (A3SS) according to the method described in Zhang et al. (2010) [24].

### GO enrichment analysis

The Fisher's exact test (N ≤ 5) or Chi-square test (N > 5) was used to calculate the significance (P-value) for each GO category. The Benjamini-Hochberg false discovery rate (FDR) was performed to adjust the P-values [55]. We used REVIGO (http://revigo.irb.hr/) to visualize enriched GO categories.

### Identification of transcription factors in *B. rapa*

Putative transcription factors in *B. rapa* were identified by BLASTP search (E-value less than 1e-5), and redundant sequences were manually discarded. The TFs domains were scanned using HMMER 3.0 software (http://hmmer.janelia.org/) using the HMM profile, which was downloaded from Pfam and PlantTFDB databases (http://arabidopsis.med.ohio-state.edu/AtTFDB/).

## Abbreviations

AS: Alternative splicing; A5SS: Alternative 5*'* splice site donor; A3SS: Alternative 3*'* splice site acceptor; B. rapa: *Brassica rapa*; ES: Exon skipping; ESTs: Expressed sequence tags; FPKM: Fragments per kilobase of exon per million fragments mapped; GO: Gene ontology; IR: Intron retention; NTRs: Novel transcribed regions; ORF: Open reading frame; RNA-seq: RNA sequencing; RT-PCR: Reverse transcription-PCR; TFs: Transcription factors; TEs: Transposable elements; UTRs: Untranslated regions; WGCNA: Weighted gene correlation network analysis.

## Competing interests

The authors declare that they have no competing interests.

## Authors’ contributions

SL and CT conceived and designed the experiments, CT, XW, JY, JW, WL, JH and CD analyzed the data, CT, WH and SL wrote the paper. All authors have read and approved the final version of the manuscript.

## Supplementary Material

Additional file 1: Figure S1 Quality for each base in reads viewed by software FastQC. **Figure S2.** Base composition of reads as visualized by the software FastQC. **Figure S3.** Evaluation of false mapping reads using duplicated genes. **Figure S4.** Genome-wide assessment of mapping RNA-seq reads. **Figure S5.** Sequencing randomness assessment. **Figure S6.** Gene coverage statistics. **Figure S7.** Comparison of gene expression in two samples of root and leaf tissues in gene expression. **Figure S8.** RT-PCR validation of novel transcripts. **Figure S9.** Cumulative distribution of FPKM values of all and novel transcripts in each sample. **Figure S10.** Expression profile of *B. rapa* genome displayed in Integrative Genomics Viewer (IGV). **Figure S11.** Numbers for different types of dinucleotides at the splicing borders. **Figure S12.** Genes with multiple AS events. Click here for file

Additional file 2: Table S1 Read mapping results under different read filtering criteria. **Table S2.** Statistic of mapping reads. **Table S3.** Constructed transcripts across all tissues. **Table S4.** Identified novel NTRs in *B. rapa*. **Table S5.** Identified 5' UTRs and 3' UTRs in *B. rapa*. **Table S6.** The uORFs in the 5' UTRs of *B. rapa* genes. **Table S7.** The FPKM values of *B. rapa* genes. **Table S8.** The 867 constitutively expressed genes whose FPKM values were greater than 50 in each tissue. **Table S9.***B. rapa* genes specifically expressed in silique and flower tissues. **Table S10.** The co-expressed gene groups (modules) from weighted gene correlation network analysis (WGCNA). **Table S11.** Numbers of identified splice junction reads in each tissue. **Table S12.** Alternative splicing events of *B. rapa* genes. **Table S13.** The enriched GO categories of *B. rapa* genes with alternative splicing events. **Table S14.** Alternative splicing events in *B. rapa* TFs genes. Click here for file
