# Supplementary material for: Comprehensive analysis of RNA-seq data reveals the complexity of the transcriptome in Brassica rapa
Source: BMC Genomics. 2013 Oct 7;14:689. doi: 10.1186/1471-2164-14-689 (PMC3853194; doi:10.1186/1471-2164-14-689)
Supplement: Additional file 1: Figure S1 — Quality for each base in reads viewed by software FastQC. Figure S2. Base composition of reads as visualized by the software FastQC. Figure S3. Evaluation of false mapping reads using duplicated genes. Figure S4. Genome-wide assessment of mapping RNA-seq reads. Figure S5. Sequencing randomness assessment. Figure S6. Gene coverage statistics. Figure S7. Comparison of gene expression in two samples of root and leaf tissues in gene expression. Figure S8. RT-PCR validation of novel transcripts. Figure S9. Cumulative distribution of FPKM values of all and novel transcripts in each sample. Figure S10. Expression profile of B. rapa genome displayed in Integrative Genomics Viewer (IGV). Figure S11. Numbers for different types of dinucleotides at the splicing borders. Figure S12. Genes with multiple AS events. [file 1471-2164-14-689-S1.doc]

**Additional file 1**

Figure S1. Quality for each base in reads viewed by software FastQC

Figure S2. Base composition of reads as visualized by the software FastQC

Figure S3. Evaluation of false mapping reads using duplicated genes

Figure S4. Genome-wide assessment of mapping RNA-seq reads

Figure S5. Sequencing randomness assessment

Figure S6. Gene coverage statistics

Figure S7. Comparison of gene expression in two samples of root and leaf tissues in gene expression

Figure S8. RT-PCR validation of novel transcripts

Figure S9. Cumulative distribution of FPKM values of all and novel transcripts in each sample

Figure S10. Expression profile of *B. rapa* genome displayed in Integrative Genomics Viewer (IGV)

Figure S11. Numbers for different types of dinucleotides at the splicing borders

Figure S12. Genes with multiple AS events

**Figure S1.** **Quality for each base in reads viewed by software FastQC.**

**Figure S2. Base composition of reads as visualized by the software FastQC.**

**
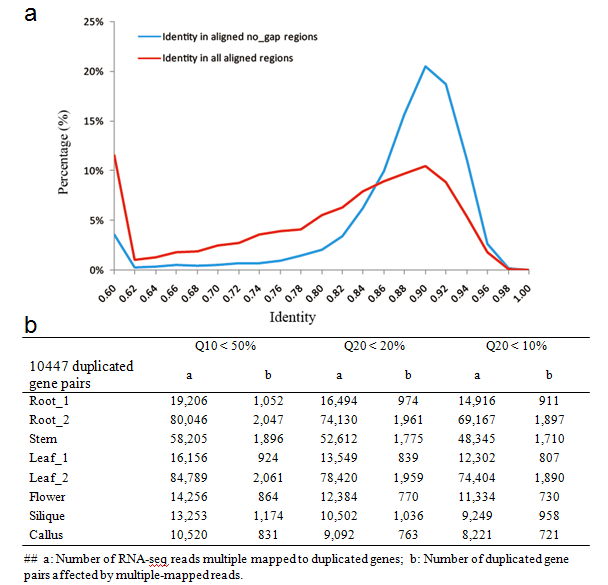
**

**Figure S3. Evaluation of false mapping reads using duplicated genes.** (a) Sequence imilarities between duplicated gene pairs (10447) retained in triplicated genomic regions of *B. rapa*. After aligning, the identity between two duplicated gene sequences were calculated according to the all aligned regions (red line) and aligned regions after removing aligned regions containing gap (-) (blue line). Sequence similarities between duplicated genes distributed over a wide range and showed a peak around 90%. Considering an at most 2 bp mis-match were allowed in read mapping, the similarity between trimmed reads and targeted reference sequences should be more than (81-2/81) = 97.53%. We observed that less than 0.2% (14) of duplicated gene pairs have similarity higher than 96%. (b) Mapping of reads to the duplicated genes retained from triplicated genomic regions. We statistic the number of reads multiple mapped to duplicated genes and the number of duplicated gene pairs affected by multiply-mapped reads (Table 4) and it shows that very few of them have been affected. Thus, the duplicated genes derived from *Brassiceae* lineage whole genome triplication (13–17 million years ago) have diverged to an extent that could be distinguishing by RNA-seq reads.

**Figure S4. Genome-wide assessment of mapping RNA-seq reads.** (A) Circle plot of the RNA-seq reads mapping to the *B. rapa* genome. The innermost depict the percentage of reads corresponding to contiguously, splicing mapped reads and unmapped reads. The middle circles depict the percentage of RNA-seq with unique/multi-mapping. The innermost circle represents the distribution of reads in exons, introns and intergenic regions according to the annotated gene set. (B) Box and whisker plots of log2-transformed average read depth per base for the following genomic regions: all intergenic sequences, introns, coding sequences, and identified novel transcripts. The three horizontal lines in the boxes show the first quantile, median and third quantile, respectively. The other two horizontal lines show the inner boundaries, and empty circles represent data outside of the inner boundaries.

**Figure S5. Sequencing randomness assessment.** The randomness of mRNA/cDNA fragmentation was evaluated with the reads distribution in reference genes. Each gene was divided into 100 windows according to the total length of coding sequences. The number of reads in each window of gene = the sum of read-depth for each base in the window / 81(bp).

**Figure S6. Gene coverage statistics.** The length percentages of reads coverage for genes were obtained. Then, the total gene numbers and percentage in each range of coverage were showed in pies.

**Figure S7.** **Comparison of gene expression in two samples of root and leaf tissues in gene expression.** The expression level of *B. rapa* annotated genes was quantified by normalized number of fragments per kilo bases per million reads (FPKM) using Cufflinks software (Trapnell, 2010 Nature Biotechnol 28, 5: 511-515). The Pearson's correlation (R value) was calculated between the log2-transformed FPKM values of two samples for leaf and root tissue, respectively.

**A)**

| **ID** | **Transcripts**  **ID** | **Primer 1**  **(5’-3’)** | **Primer 2**  **(5’-3’)** | **Size (bp)** | **Scaffold** | **Start** | **End** |
| --- | --- | --- | --- | --- | --- | --- | --- |
| 1 | CUFF.35943 | AGTAACTTTCAATCTTGCCTCT | GGCGGAGTAAACAACAAATA | 159 | Scaffold000170 | 259523 | 259934 |
| 2 | CUFF.22518 | GCCAGTGAGTGAGCATAGGG | TGTGCCAAGGAGCTTTAGAC | 305 | A07 | 10371787 | 10373240 |
| 3 | CUFF.7947 | GTATTGGCTTACTGGCAGATGG | GAAAGGTCCGAGTTTGGTGG | 496 | A03 | 5018474 | 5020442 |
| 4 | CUFF.16809 | TGCATAGCGAGCAAACAGAC | GGGTGGGCGACTTCTAATGT | 312 | A05 | 19809444 | 19811671 |
| 5 | CUFF.25971 | GTTTACCGAACCCGAAGTCT | CGTGGAGCAAATACAGGAGG | 362 | A08 | 10262976 | 10263810 |
| 6 | CUFF.36671 | GATGAGCCTTTGATTAGAACTT | GTTGTCCTGGTCTGTCCCTA | 310 | Scaffold004752 | 166 | 420 |

**B)**


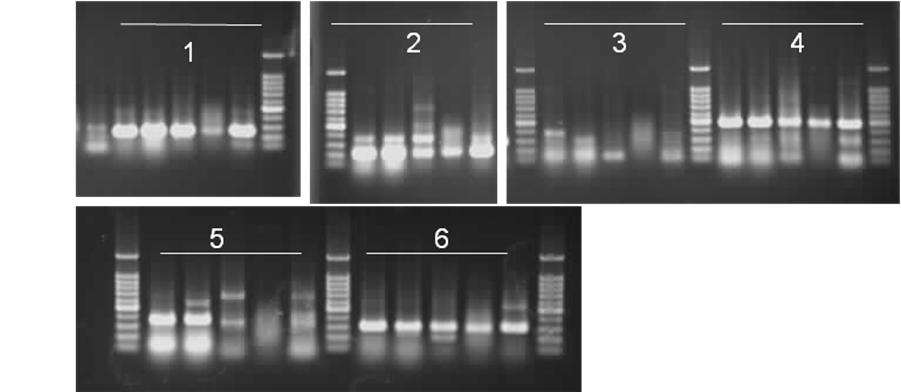


**Figure S8. RT-PCR validation of novel transcripts. (A)** The primers designed for 6 randomly selected novel transcripts. **(B)** The RT-PCR results of amplification of 6 (ID 1-6 in this picture) randomly selected novel transcripts predicted by Cufflink in this paper. Each of six Cufflink-predicted transcripts was amplified in five tissues: root, stem, leaf, flower and silique (from left to right in panel for each transcript). The primers for amplifying the selected novel cufflink TU in this paper are as follows.

**Figure S9. Cumulative distribution of FPKM values of all and novel transcripts in each sample.** The P-value was calculated from Wilcoxon test for annotated novel transcripts (red line) and all identified transcripts (green line) in each tissue.

**
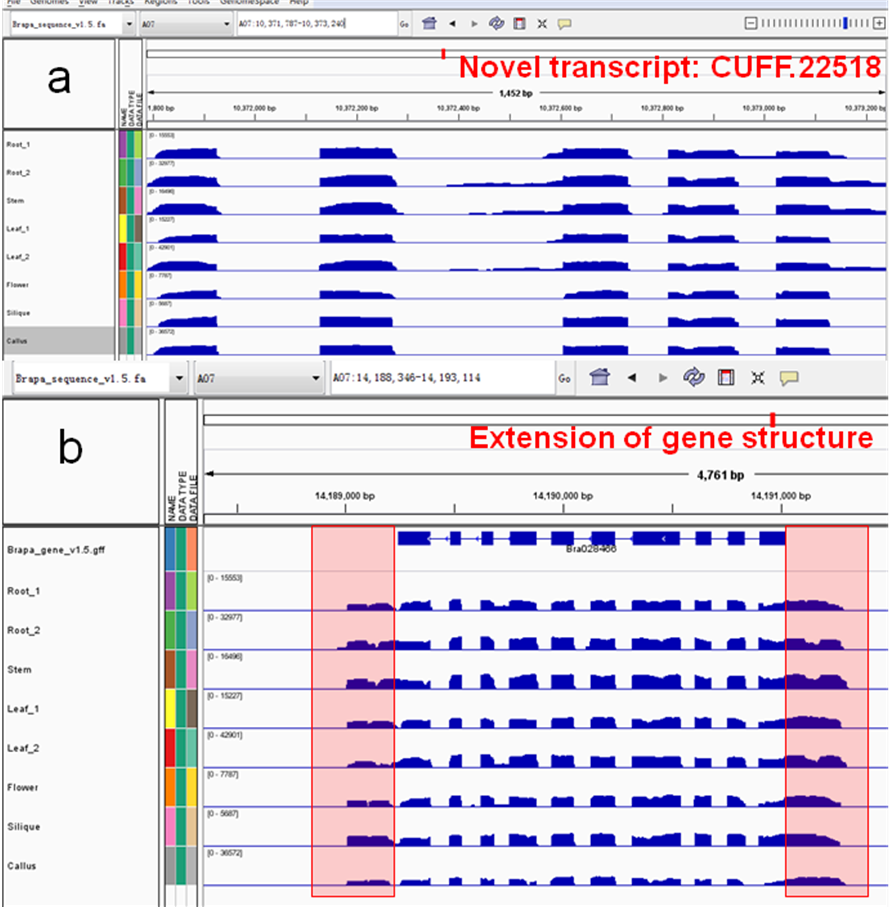
**

**Figure S10. Expression profile of *B. rapa* genome displayed in Integrative Genomics Viewer (IGV). (A)** An identified novel transcribed regions (for example), which was not annotated in *B. rapa* chromosome. **(B)** The tissue conserved expression level and the extension of gene structure (the peak for each base) shown for a gene (Bra028466) in A07 chromosome of *B. rapa*.

**Figure S11. Numbers for different types of dinucleotides at the splicing borders.**

**Figure S12.** **Genes with multiple AS events.** The gene number (y-axis) for gene with different AS events number (x-axis).
